# Transmitted Antiretroviral Drug Resistance in New York State, 2006-2008: Results from a New Surveillance System

**DOI:** 10.1371/journal.pone.0040533

**Published:** 2012-08-06

**Authors:** Adam C. Readhead, Daniel E. Gordon, Zhengyan Wang, Bridget J. Anderson, Kathleen S. Brousseau, Maria A. Kouznetsova, Lisa A. Forgione, Lou C. Smith, Lucia V. Torian

**Affiliations:** 1 The New York City Department of Health and Mental Hygiene, HIV Epidemiology and Field Services Program, New York, New York, United States of America; 2 The New York State Department of Health, Bureau of HIV/AIDS Epidemiology, Albany, New York, United States of America; Institut National de la Santé et de la Recherche Médicale, France

## Abstract

**Background:**

HIV transmitted drug resistance (TDR) is a public health concern because it has the potential to compromise antiretroviral therapy (ART) at the population level. In New York State, high prevalence of TDR in a local cohort and a multiclass resistant case cluster led to the development and implementation of a statewide resistance surveillance system.

**Methodology:**

We conducted a cross-sectional analysis of the 13,109 cases of HIV infection that were newly diagnosed and reported in New York State between 2006 and 2008, including 4,155 with HIV genotypes drawn within 3 months of initial diagnosis and electronically reported to the new resistance surveillance system. We assessed compliance with DHHS recommendations for genotypic resistance testing and estimated TDR among new HIV diagnoses.

**Principal Findings:**

Of 13,109 new HIV diagnoses, 9,785 (75%) had laboratory evidence of utilization of HIV-related medical care, and 4,155 (43%) had a genotype performed within 3 months of initial diagnosis. Of these, 11.2% (95% confidence interval [CI], 10.2%–12.1%) had any evidence of TDR. The proportion with mutations associated with any antiretroviral agent in the NNRTI, NRTI or PI class was 6.3% (5.5%–7.0%), 4.3% (3.6%–4.9%) and 2.9% (2.4%–3.4%), respectively. Multiclass resistance was observed in <1%. TDR did not increase significantly over time (p for trend = 0.204). Men who have sex with men were not more likely to have TDR than persons with heterosexual risk factor (OR 1.0 (0.77–1.30)). TDR to EFV+TDF+FTC and LPV/r+TDF+FTC regimens was 7.1% (6.3%–7.9%) and 1.4% (1.0%–1.8%), respectively.

**Conclusions/Significance:**

TDR appears to be evenly distributed and stable among new HIV diagnoses in New York State; multiclass TDR is rare. Less than half of new diagnoses initiating care received a genotype per DHHS guidelines.

## Introduction

The widespread use of anti-retroviral therapy (ART) and the extended survival of HIV-infected individuals have produced a growing population of ART-experienced persons who may develop antiretroviral (ARV) drug resistance. Individuals with ARV resistance have reduced responsiveness to ART, delayed or incomplete viral suppression and poor outcomes [1], [2]. Moreover, they may transmit resistant infection to others. Transmitted drug resistance (TDR) is a public health concern because it has the potential to compromise ART at the population level. In New York State, a report of increasing TDR in a local cohort [3] and a case cluster involving transmission of a multi-class resistant virus [4]–[6] suggested the need to monitor TDR statewide. In 2005, building on existing HIV surveillance, which already included routine reporting of viral loads, CD4 counts and positive Western blots, [7]–[10] New York State introduced mandatory electronic reporting of viral nucleotide sequences for the purpose of conducting resistance surveillance [11], [12]. We report results of the first three years of data from the New York State resistance surveillance system, the first of its kind in the U.S.

## Methods

### Data Sources

The HIV/AIDS surveillance systems of the New York State Department of Health (NYSDOH) and the New York City Department of Health and Mental Hygiene (NYC DOHMH) have been described previously [13]–[15]. Nucleotide sequences from HIV genotypes, along with other HIV-related tests and conditions, are reportable by law [7]–[12]. Laboratory and provider reports are transmitted to NYSDOH where they are matched to the New York State HIV registry; data relating to cases within New York City are forwarded to NYC DOHMH where they are matched to the NYC HIV registry. Incoming data at the state or city level that do not match an existing registry record initiate a field investigation to confirm the case, date and disposition of diagnosis and collect other data required by surveillance. An analysis dataset was created based on diagnoses and laboratory results dated January 1, 2006, through December 31, 2008, reported by April 30, 2010, and added to the NYS HIV registry as of May 31, 2010. A total of 14,046 persons aged 13 and older and not perinatally infected had an initial diagnosis date between January 1, 2006, and December 31, 2008; 937 (6.7%) were excluded because of missing or discrepant data on date of initial diagnosis or genotype, leaving 13,109 for analysis.

**Table 1:** Frequency of ARV drug resistance testing within 3 months of HIV diagnosis, New York State 2006–2008†.

	Newly-diagnosed HIV cases	Cases in care**		Cases with initial genotype test					Multivariate Logistic Regression	
	Total N	N	as % of total	N	as % of new diagnoses	as % of cases in care	Crude OR (95% CI)	P	Adjusted OR (95% CI) (n = 8,074)*	P
All	13109	9785	74.6	4155	31.7	42.5				
Sex								<.0001		0.3046
Male	9467	7002	74.0	3090	32.6	44.1	Referent		Referent	
Female	3642	2783	76.4	1065	29.2	38.3	0.78 (0.72–0.86)		1.07 (0.94–1.20)	
Race/Ethnicity								<.0001		<.0001
Black	6177	4439	71.9	1671	27.1	37.6	0.61 (0.55–0.67)		0.70 (0.61–0.79)	
Hispanic	3804	2861	75.2	1232	32.4	43.1	0.76 (0.68–0.85)		0.85 (0.74–0.97)	
White	2539	2027	79.8	1011	39.8	49.9	Referent		Referent	
Asian/Pacific Islander	286	214	74.8	127	44.4	59.3	1.47 (1.10–1.95)		1.35 (0.98–1.85)	
Native American/Multirace	303	244	80.5	114	37.6	46.7	0.88 (0.68–1.15)		0.87 (0.65–1.16)	
Age at Diagnosis								<.0001		0.0010
13–24	2140	1469	68.6	557	26.0	37.9	0.75 (0.66–0.85)		0.77 (0.67–0.89)	
25–39	5310	3920	73.8	1758	33.1	44.8	Referent		Referent	
40–59	5079	3917	77.1	1630	32.1	41.6	0.88 (0.80–0.96)		0.87 (0.79–0.97)	
60+	580	479	82.6	210	36.2	43.8	0.96 (0.79–1.16)		0.96 (0.78–1.19)	
Risk								<.0001		<.0001
MSM (+MSM/IDU)	5499	4136	75.2	1977	36.0	47.8	Referent		Referent	
IDU	785	560	71.3	197	25.1	35.2	0.59 (0.49–0.71)		0.63 (0.51–0.78)	
Heterosexual	2225	1718	77.2	696	31.3	40.5	0.74 (0.66–0.83)		0.77 (0.66–0.90)	
No Identified Risk	4600	3371	73.3	1285	27.9	38.1	0.67 (0.61–0.74)		0.69 (0.61–0.78)	
Residence at diagnosis								<.0001		0.0002
City	10412	7639	73.4	3119	30.0	40.8	Referent		Referent	
Rest of State	2697	2146	79.6	1036	38.4	48.3	1.35 (1.23–1.49)		1.25 (1.11–1.40)	
Poverty								<.0001		0.1700
Non-poverty Area	5967	4577	76.7	2108	35.3	46.1	Referent		Referent	
Poverty Area	6851	5026	73.4	1979	28.9	39.4	0.76 (0.70–0.82)		0.93 (0.85–1.03)	
Missing zip	291	182	62.5	68	23.4	37.4				
Year of diagnosis								<.0001		<.0001
2006	4496	3265	72.6	1132	25.2	34.7	Referent		Referent	
2007	4382	3224	73.6	1404	32.0	43.5	1.45 (1.31–1.61)		1.46 (1.31–1.63)	
2008	4231	3296	77.9	1619	38.3	49.1	1.82 (1.65–2.01)		1.85 (1.66–2.06)	
Clinical stage at diagnosis										
HIV only	9553	6392	66.9	2481	26.0	38.8				
HIV/AIDS	3556	3393	95.4	1674	47.1	49.3				
CD4 count								<.0001		<.0001
<350	4932	4932	100.0	2453	49.7	49.7	Referent		Referent	
> = 350	4156	4156	100.0	1489	35.8	35.8	0.57 (0.52–0.62)		0.57 (0.51–0.62)	
Missing CD4	4021	697	17.3	213	3.8	30.1				
VL								<.0001		<.0001
<10,000	2904	2904	100.0	922	31.7	31.7	0.10 (0.07–0.13)		0.14 (0.10–0.19)	
10,000–100,000	3425	3425	100.0	1658	48.4	48.4	0.69 (0.63–0.75)		0.85 (0.77–0.95)	
> = 100,000	2493	2493	100.0	1323	53.1	53.1	Referent		Referent	
Missing VL	4287	963	22.5	252	5.9	26.2	0.31 (0.27–0.37)		0.32 (0.27–0.39)	

† Excludes persons aged 12 years or younger and persons perinatally infected.

* Multivariate logistic regression excludes cases with missing data except for missing VL and missing risk which is categorized as “No identifiable risk”.

** Represents total after removal of 3324 cases missing CD4, VL and/or genotype within three months of diagnosis.

**Table 2:** Frequency of TDR genotypes by demographic characteristics, New York State 2006–2008.

	Resistant to 1 or more ARVs		Cases with full analyzable sequence			Multivariate logistic regression (n = 3,791)*	
	N	%	N	Crude OR (95% CI)	P	Adjusted OR (95% CI)	P
All	450	11.2	4032				
Sex					0.679		
Male	332	11.0	3007	Referent			
Female	118	11.5	1025	1.05 (0.84–1.31)			
Race/Ethnicity					0.346		
Black	164	10.1	1620	0.78 (0.61–1.01)			
Hispanic	140	11.7	1198	0.92 (0.71–1.19)			
White	123	12.6	979	Referent			
Asian/Pacific Islander	12	9.7	124	0.75 (0.40–1.39)			
Native American/Multirace	11	9.9	111	0.77 (0.40–1.47)			
Age at Diagnosis					0.014		0.078
13–24	78	14.3	544	1.26 (0.95–1.67)		1.22 (0.91–1.61)	
25–39	199	11.7	1698	Referent		Referent	
40–59	150	9.5	1584	0.79 (0.63–0.99)		0.83 (0.66–1.04)	
60+	23	11.2	206	0.95 (0.60–1.50)		1.06 (0.66–1.69)	
Risk					0.002		0.010
MSM (+MSM/IDU)	242	12.6	1922	Referent		Referent	
IDU	17	8.7	195	0.66 (0.40–1.11)		0.74 (0.44–1.25)	
Heterosexual	85	12.6	676	1.00 (0.77–1.30)		1.03 (0.79–1.35)	
No Identified Risk	106	8.6	1239	0.65 (0.51–0.83)		0.68 (0.53–0.87)	
Residence at diagnosis					0.905		
City	317	11.1	2850	Referent			
Rest of State	133	11.3	1182	1.01 (0.82–1.26)			
Poverty					0.770		
Non-poverty Area	228	11.1	2052	Referent			
Poverty Area	207	10.8	1913	0.97 (0.80–1.18)			
Missing zip	15	22.4	67				
Year of diagnosis					0.011		0.022
2006	123	11.5	1073	Referent		Referent	
2007	123	9.2	1344	0.78 (0.60–1.01)		0.79 (0.60–1.03)	
2008	204	12.6	1615	1.12 (0.88–1.42)		1.10 (0.86–1.40)	
Clinical stage at diagnosis							
HIV only	281	11.7	2401				
HIV/AIDS	169	10.4	1631				
CD4 count					0.217		
<350	253	10.6	2389	Referent			
> = 350	171	11.9	1439	1.14 (0.93–1.40)			
Missing CD4	26	12.7	204				
VL					0.593		0.765
<10,000	124	13.9	892	1.61 (0.74–3.49)		1.50 (0.69–3.29)	
10,000–100,000	164	10.2	1607	1.09 (0.88–1.35)		1.02 (0.82–1.27)	
> = 100,000	137	10.6	1292	Referent		Referent	
Missing VL	25	10.4	241	0.98 (0.62–1.53)		0.95 (0.60–1.50)	

* Multivariate logistic regression excludes cases with missing data except for missing risk which is categorized at “No identifiable risk”.

### Data definitions

Diagnosis refers to a new diagnosis of HIV with or without a concurrent diagnosis of AIDS. Concurrent diagnosis was defined as AIDS diagnosis within 31 days of initial diagnosis of HIV. Region at diagnosis was categorized as New York City or New York State excluding New York City. Poverty area was defined as residence at diagnosis in a ZIP code tabulation area in which at least 20% of residents per US Census 2000 met the federal definition of poverty. Poverty area was not calculated for homeless or sheltered persons or for persons residing in zip codes created after 2000. Cases with missing risk factor were assigned to the category, “no identified risk.” Initial resistance test was defined as the first HIV genotype (if any) within 3 months of diagnosis. The 3 month interval was chosen to limit the number of persons that may have started ART before resistance testing and to allow comparison with results from the Centers for Disease Control's (CDC) Variant, Atypical, and Resistant HIV Surveillance (VARHS) system [16]–[17]. In addition to nucleotide sequences, laboratory data included the first CD4 count and viral load drawn within 3 months of diagnosis. Persons with a viral load, CD4 count or resistance test within 3 months of diagnosis were considered in care because these tests must be ordered by a physician [14]. CD4 counts were dichotomized as >350 cells/ml or <350 cells/ml because Department of Health and Human Services (DHHS) guidelines in place during the reporting period recommended initiation of ART at this threshold [18]. Viral loads were grouped into three intervals, <10,000 copies/ml, 10,000–100,000 copies/ml, and >100,000 copies/ml.

**Table 3:** Transmitted Drug Resistance by drug class. New York State 2006–2008.

	Total			
	N	%	95% CI	
Genotypes Analyzed	4032	-		
Any	450	11.2%	10.2%	12.1%
NRTI	172	4.3%	3.6%	4.9%
NNRTI	252	6.3%	5.5%	7.0%
PI	116	2.9%	2.4%	3.4%
Two Class				
NRTI-NNRTI	33	0.8%	0.5%	1.1%
NRTI-PI	15	0.4%	0.2%	0.6%
NNRTI-PI	16	0.4%	0.2%	0.6%
Three Class				
NRTI-NNRTI-PI	13	0.3%	0.1%	0.5%

**Table 4:** Top 10 mutations by drug class.

rank	NRTI Mutation	NNRTI Mutation	PI Mutation
1	G333E	K103N	L10I
2	V118I	E138A	A71T
3	M41L	K103R	L10V
4	T69N	V179D	A71V
5	G333D	K101Q	L10IL
6	T215D	G190A	L90M
7	G333EG	V179E	A71AT
8	M184V	V108I	T74S
9	D67N	K103KR	A71AV
10	V118IV	Y181C	V11I

### Resistance Analysis

HIV genotype testing was performed by commercial laboratories using various test kits, including Viroseq™, GenoSure™, TRUGENE™ and in-house kits. Only protease and reverse transcriptase sequences of the *pol* gene were reported. Nucleotide sequences were analyzed using the Resistance Analysis System (RAS), version 2.0 (Frontier Science & Technology Research Foundation, Amherst, New York), a program built specifically to facilitate NYS resistance surveillance. Mutations were ascertained by a comparison of aligned sequences with the Los Alamos National Laboratory subtype B consensus sequence [19]. ARV-specific predicted resistance was calculated using code developed by Frontier Science and scores from the Stanford HIVDB algorithm, version 6.0.9; [20], [21] this algorithm was also used to determine HIV-1 subtype. Sequences that did not meet the minimum processing requirement of the HIVDB algorithm could not be analyzed [20].

Transmitted drug resistance was defined as the presence of 1 or more mutations in the surveillance drug resistance mutation list (SDRM) [22]. ARVs were categorized by class. Single, double or triple class resistance was defined as 1 or more surveillance drug mutation within one, two or three antiretroviral drug classes respectively. Predicted resistance to specific antiretroviral drugs was defined as sequences with a score of ≥4 on the Stanford HIVDB 5-point resistance scale [20].

**Figure 1:**
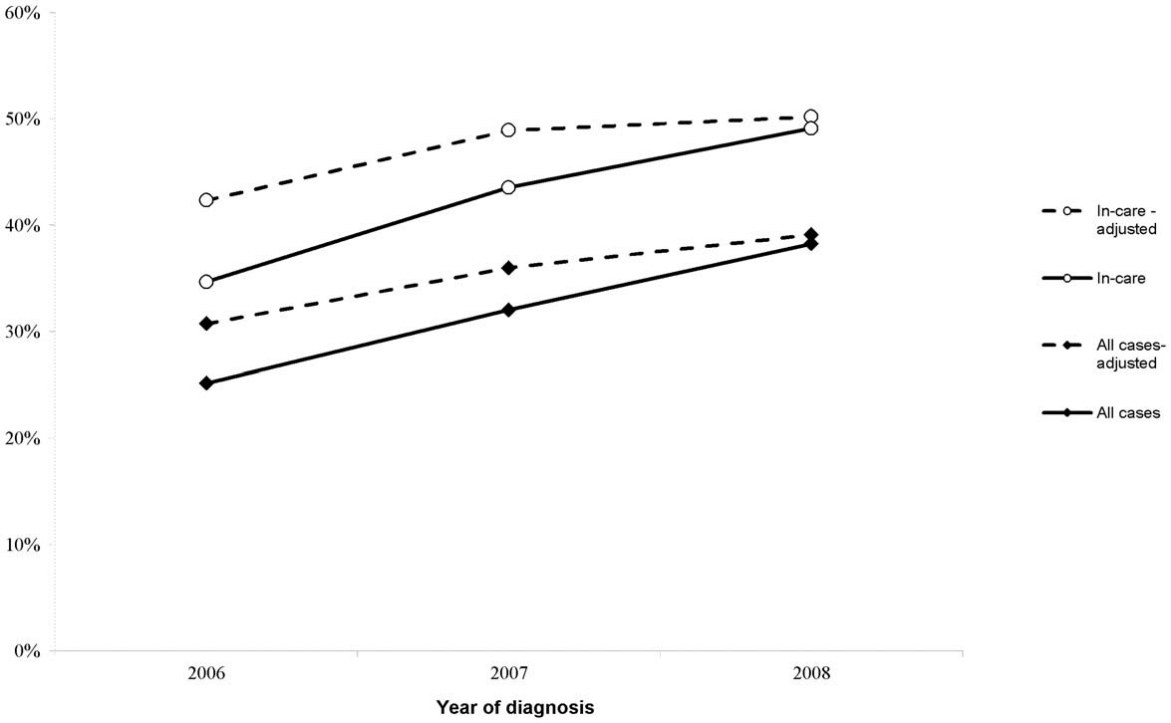
Proportion of cases receiving genotype within 3 months of diagnosis, with adjustment for completeness of reporting.

### Statistical Analysis

Multivariate logistic regression was used to assess the likelihood of an initial resistance test and the likelihood of TDR as a function of demographic and clinical characteristics. Unadjusted and adjusted odd ratios with 95% confidence intervals (CIs) were calculated. Concurrent diagnosis of HIV/AIDS was excluded from the regression analysis of testing patterns because it is partially defined by CD4 count. Variables significant (p<0.05) on bivariate analysis were entered into multivariate logistic regression models for the two outcome variables, testing and TDR. Confidence limits for proportions were calculated using exact CIs for the binomial proportion. Trends were examined using the Cochran-Armitage test and are reported with two-sided p-values. All statistical tests were performed using SAS, version 9.1.3 (SAS Institute, Cary, North Carolina).

## Results

### Population Demographics and Resistance Testing Patterns

Of the 13,109 persons included in the analysis, 4,155 (31.7%) received their first resistance test within 3 months of diagnosis (“initial resistance test”); 1,311 (10.0%) were first genotyped >3–12 months after diagnosis, 7,643 (13.3%) were first genotyped >12 months after diagnosis, and 44.9% were never genotyped. Of all persons ever genotyped, three-quarters were genotyped within three months of initial diagnosis. Patients never genotyped differed significantly from patients ever genotyped by age, race, risk factor, and disease stage at diagnosis (data not shown). Patients with CD4<350, VL>100,000 and concurrent HIV/AIDS at diagnosis, i.e., patients meeting DHHS guidelines for ART, were more likely to have ever been genotyped.

Initial resistance testing among newly diagnosed persons differed significantly by sex, race/ethnicity, age, risk factor, region of diagnosis, poverty area, year of diagnosis, and disease stage at diagnosis (Table 1). Among persons with new diagnoses, 9,785 (74.6%) showed evidence of care (i.e., saw a physician) within 3 months of diagnosis. Of persons in care, 4,155 (43%) had initial resistance tests. Among all newly diagnosed, the proportion with an initial resistance test increased from 25% in 2006 to 38% in 2008 (p for trend <0.0001). Subsequent analyses were conducted among persons in care within three months (N = 9,785 or 74.6% of the total number of newly diagnosed) because these would be the only persons in the database who would have had the opportunity for initial resistance testing.

In the multivariate analysis of initial resistance testing among newly diagnosed persons in care, blacks and Hispanics were less likely to be tested than whites (AOR 0.70 (0.61–0.79), AOR 0.85 (0.74–0.97)) (Table 1). Persons aged 13–24 or 40–59 at diagnosis were slightly less likely to be tested than those 25–39 (AOR 0.77 (0.67–0.89); AOR 0.87 (0.79–0.97)), while persons 60 and older were no more likely to be tested (AOR 0.96 (0.79–1.19)). Compared with men who have sex with men (including men who have sex with men and use injection drugs (MSM + MSM/IDU)), persons with heterosexual transmission risk were less likely to be tested (AOR 0.77 (0.66–0.90)).

Persons diagnosed in New York State excluding New York City were more likely to have a resistance test than persons diagnosed in New York City (AOR 1.25 (1.11–1.40)), as were persons diagnosed in 2008 in comparison to those diagnosed in 2006 (AOR 1.85 (1.66–2.06)). Persons living in a non-poverty area were not significantly more likely to have a resistance test than those living in a poverty area (AOR 0.93 (0.85–1.03)). Persons with initial CD4 count ≥350 cells/ml were less likely to have a resistance test than persons with CD4 count <350 cells/ml (AOR 0.57 (0.51–0.62)), and persons with viral loads of <10,000 copies/mL or 10,000–100,000 copies/mL were less likely to have a resistance test than persons with >100,000 copies/mL (AOR 0.14 (0.10–0.19), AOR 0.85 (0.77–0.95)).

### Resistance patterns

Of the 4,155 initial resistance tests, 123 were reported with partial nucleotide sequences, and 4,032 (97.0%) had analyzable sequences. Among these, 450 (11.2% (10.2%–12.1%)) had evidence of TDR (Table 2). TDR did not significantly increase over time (p for trend = 0.204). In the multivariate analysis, risk, year of diagnosis and viral load remained significantly associated with TDR., However persons with a heterosexual risk factor were no more likely to have resistance than MSM (AOR 1.03 (0.79–1.35)). In addition, black and Hispanic MSM were no more likely to have TDR in comparison with other race and risk groups (OR 1.10 (0.82–1.49) data not shown). Persons with no identified risk (NIR) were significantly less likely to have TDR than MSM (AOR 0.68 (0.53–0.87)).

TDR varied by drug class. Resistance was highest to NNRTIs (6.3% (5.5%–7.0%)) and was significantly higher than resistance to NRTIs (4.3% (3.6%–4.9%)) and PIs (2.9% (2.4%–3.4%)) (Table 3). Over time, there was no significant increase in resistance in the NNRTI, NRTI or PI classes (p = 0.144, p = 0.686, p = 0.851, respectively). Resistance in two classes was highest in the NRTI-NNRTI (0.8%) combination; resistance in three classes was minimal (<0.5%). The most frequently observed polymorphisms by drug class are shown in Table 4.

We also examined predicted resistance to 1 or more components of selected starting regimens recommended by DHHS [18]. Resistance to 1 or more ARVs within the NNRTI-inclusive regimen EFV+TDF+FTC was observed in 7.1% (6.3%–7.9%) of cases (data not shown). In contrast, 1.4% (1.0%–1.8%) were resistant to 1 or more drugs in the PI-inclusive regimen LPV/r+TDF+FTC. Among individual NRTIs, resistance was highest to AZT and D4T (1.8% (1.4%–2.2%) and 1.6% (1.2%–2.0%)) and lower to TDF (0.3% (0.1%–0.5%) and FTC (0.9% (0.6%–1.2%), the two agents with recently demonstrated efficacy in pre-exposure prophylaxis [23]. 3TC, which in the treatment setting can be used interchangeably with FTC, showed similarly low resistance (0.9% (0.6%–1.2%)).

Most analyzed sequences (92.8%) were subtype B; 118 (2.9%) were CRF02_AG; and 83 (2.1%) were subtype C. Persons residing in NYC at diagnosis were no more likely to have non-B subtypes than persons residing in New York State excluding New York City (7.7% vs. 6.0%, p = 0.0611).

## Discussion

### Resistance Testing Patterns

Within the U. S., this analysis represents the first use of routinely reported surveillance data to estimate TDR and to describe resistance testing patterns as well as the largest number of sequences used for resistance surveillance to date [16], [24]. More than half of newly diagnosed persons who entered care within three months did not receive an initial resistance test per DHHS guidelines, although the proportion receiving initial resistance tests increased between 2006 and 2008. The observed increase is consistent with the adoption of the 2007 DHHS guidelines recommending resistance testing for all newly diagnosed persons [18], [25]. Previous guidelines recommended resistance testing for acute infection and patients initiating or failing ART [23]. Significant differences in resistance testing by demographic characteristics, including race, age and transmission risk, are concordant with literature on initiation, source, and utilization of care [26]–[28]. Potential candidates for initiation of ART per DHHS guidelines (CD4<350) were more likely to be tested than others, likely reflecting the decision by some providers to postpone resistance testing until initiation of ART. Similarly, persons with low viral loads (<10,000) were less likely to be tested. While this could be evidence of the impact of viral load on a provider's decision to genotype, it may also be affected by the failure of amplification and genotyping at low viral loads (failed genotypes are not reported). Resistance testing was less common among NYC residents than residents in the rest of the state, an unexpected finding given the concentration of training hospitals and designated AIDS centers in the city. Further analysis is needed to elucidate the relationship between resistance testing, provider type, and utilization of care.

### Transmitted Drug Resistance

The prevalence of TDR among persons with new diagnoses in NYS in 2006–2008 was 11.2% (10.2%–12.1%). There was no significant change in TDR over time. Worldwide estimates of TDR range from 8%–24%, though comparison between these results is difficult due to differences in the mutations used to define TDR [29]–[38]. Our estimate, based on the SDRM list [22], is higher than the national prevalence estimate (8.3%) for the time period 1997–2001 [39] but is substantially lower than a previous report of resistance in a NYC sample of MSM in 2003–2004 (24.1%) [3]. Both of these studies used modified IAS-USA mutation lists. Wheeler et al. estimated the national prevalence of transmitted drug resistant mutations (TDRM) in 2006 to be 14.6% using a modified SDRM list [16]. We estimated the New York State TDR to be 24.2% using the same mutation list (results not shown). Further analysis is needed to test the utility of the SDRM and TDRM lists in the U.S. epidemic.

In contrast to previous findings of increasing TDR and high levels of TDR among MSM, we found stable resistance evenly distributed between MSM and heterosexual risk groups [3], [40]. Better risk factor ascertainment would allow us to measure the TDR by risk factor more accurately and/or to understand the unexpected findings of this analysis. Our data show that 1 in 9 persons newly diagnosed with HIV in NYS has TDR and 1 in 50 is predicted to have a suboptimal response to a standard ART regimen. Key populations considered to be on the leading edge of the epidemic, e.g., young black and Hispanic MSM, showed no more TDR than others. Ongoing surveillance will confirm the significance and durability of these observations.

### Limitations

Our analysis has important limitations. HIV surveillance data contain limited person-level information; duration of infection and ART history are not available. Newly-diagnosed persons are assumed to be ARV-naïve but may not be. Despite the CDC-sponsored routine interstate duplication review (RIDR) and comprehensive field investigation, persons may be incorrectly identified as newly diagnosed because there is incomplete date information or because they were diagnosed out of state and subsequently received HIV care in New York State. In such cases acquired resistance may be incorrectly classified as TDR. The number of resistance tests reported is an underestimate of the number ordered by providers because resistance tests in which viral RNA amplification fails are not reportable.

Integrating resistance data into the existing surveillance system was logistically and technically challenging. Laboratories certified by NYSDOH to perform resistance testing were required to report nucleotide sequences beginning on June 1, 2005. However, laboratories acquired full capacity to report resistance data at different times after the regulations were enacted, which meant that much of the data was reported retrospectively. Laboratories were required to resubmit when incomplete data were identified; however, some laboratories were not able to do so. Completeness of laboratory reporting was estimated by comparing self-reported laboratory testing logs to received data transmissions. Completeness was estimated to be 82% in 2006, 89% in 2007, and 98% in 2008. Adjusting for completeness, the proportion of persons with new diagnoses with initial resistance tests increased from 29% in 2006 to 39% in 2008 (p<.0001) (Figure 1). Incomplete data in key fields (e.g. name and date of birth) affected the matching of some reports to the surveillance registry. However, the proportion of resistance tests that could be matched was similar to other reportable tests.

The completeness and accuracy of risk ascertainment is an ongoing challenge for surveillance. Misclassification of heterosexual transmission as NIR and misclassification of MSM as heterosexual may account for our observations of reduced risk associated with NIR and equivalent risk in MSM and heterosexuals [41].

Our TDR estimate may be biased because it is based on genotypes for less than one-third of persons with new diagnoses and less than one-half of those initiating care within 3 months. It is possible that TDR in persons not genotyped is significantly different from the TDR patterns reported here. In addition, our estimate may under-represent clinically important resistance; minority quasispecies, not detectable by genotypes reported to NYSDOH, have been shown to be prevalent in untreated persons and to reduce treatment efficacy [42]. Finally, current reporting does yet not allow the monitoring of resistance certain classes of ARVs including integrase strand-transfer inhibitors, entry inhibitors or CCR5 receptor antagonists.

### Conclusion

Using the New York State HIV resistance surveillance system, we have taken an important step in addressing transmitted drug resistance as a public health concern. In contrast to earlier local reports, our data suggest that TDR is not increasing and that multiclass TDR is not prevalent. Furthermore, TDR is not isolated to a specific subgroup, and common starting regimens are still effective for most new diagnoses in New York State. This information will help shape our response to the epidemic in both the public health and medical communities.

This analysis suggests that continuing routine resistance surveillance is appropriate for three reasons. First, more data are needed to verify the trend in TDR. Resistance surveillance systems such as the one describe here are uniquely qualified to provide consistent, long-term monitoring. Methodological differences between short-term studies make it difficult to evaluate trends in TDR. Second, treatment-intensive community strategies such as ‘Test and Treat’ and PrEP may increase TDR. Third, in contrast to surveillance based on specimen salvage, which is costly and logistically difficult, resistance surveillance through routine electronic reporting is relatively low cost and scalable. If improved TDR estimation is found to be necessary, routine reporting could be supplemented with specimen salvage from new diagnoses without routine genotype results.

This work illustrates the power of surveillance to establish baselines and monitor progress toward goals established to achieve epidemic mitigation and control [43]. However, broader provider uptake of genotype testing is needed to better estimate population TDR and to understand the TDR prevalence at which routine genotyping and surveillance of new diagnoses provide clinically and epidemiologically significant information.
